# Determinants of Follow-Up Participation in the Internet-Based European Influenza Surveillance Platform Influenzanet

**DOI:** 10.2196/jmir.3010

**Published:** 2014-03-10

**Authors:** Paolo Bajardi, Alessandro Vespignani, Sebastian Funk, Ken TD Eames, W John Edmunds, Clément Turbelin, Marion Debin, Vittoria Colizza, Ronald Smallenburg, Carl E Koppeschaar, Ana O Franco, Vitor Faustino, Annasara Carnahan, Moa Rehn, Daniela Paolotti

**Affiliations:** ^1^GECO - Computational Epidemiology GroupDepartment of Veterinary SciencesUniversity of TorinoTorinoItaly; ^2^Complex Systems UnitMolecular Biotechnology Center CSUUniversity of TorinoTorinoItaly; ^3^Institute for Scientific Interchange FoundationTorinoItaly; ^4^Laboratory for the Modeling of Biological and Socio-Technical SystemsNortheastern UniversityBoston, MAUnited States; ^5^Institute for Quantitative Social SciencesHarvard UniversityCambridge, MAUnited States; ^6^London School of Hygiene and Tropical MedicineLondonUnited Kingdom; ^7^INSERMUMR-S 1136Institut Pierre Louis d’Epidémiologie et de Santé Publique, F-75013ParisFrance; ^8^Sorbonne UniversitésUPMC Univ Paris 06, UMR-S 1136Institut Pierre Louis d’Epidémiologie et de Santé Publique, F-75013ParisFrance; ^9^Science in Action BVAmsterdamNetherlands; ^10^Instituto Gulbenkian de CiênciaOeirasPortugal; ^11^Public Health Agency of SwedenStockholmSweden

**Keywords:** participatory surveillance, Internet, influenza

## Abstract

**Background:**

“Influenzanet” is a network of Internet-based platforms aimed at collecting real-time data for influenza surveillance in several European countries. More than 30,000 European volunteers participate every year in the study, representing one of the largest existing Internet-based multicenter cohorts. Each week during the influenza season, participants are asked to report their symptoms (if any) along with a set of additional questions.

**Objective:**

Focusing on the first influenza season of 2011-12, when the Influenzanet system was completely harmonized within a common framework in Sweden, the United Kingdom, the Netherlands, Belgium, France, Italy, and Portugal, we investigated the propensity of users to regularly come back to the platform to provide information about their health status. Our purpose was to investigate demographic and behavioral factors associated with participation in follow-up.

**Methods:**

By means of a multilevel analysis, we evaluated the association between regular participation during the season and sociodemographic and behavioral characteristics as measured by a background questionnaire completed by participants on registration.

**Results:**

We found that lower participation in follow-up was associated with lower educational status (odds ratio [OR] 0.80, 95% CI 0.75-0.85), smoking (OR 0.64, 95% CI 0.59-0.70), younger age (OR ranging from 0.30, 95% CI 0.26-0.33 to 0.70, 95% CI 0.64-0.77), not being vaccinated against seasonal influenza (OR 0.77, 95% CI 0.72-0.84), and living in a household with children (OR 0.69, 95% CI 0.65-0.74). Most of these results hold when single countries are analyzed separately.

**Conclusions:**

Given the opportunistic enrollment of self-selected volunteers in the Influenzanet study, we have investigated how sociodemographic and behavioral characteristics may be associated with follow-up participation in the Influenzanet cohort. The study described in this paper shows that, overall, the most important determinants of participation are related to education and lifestyle: smoking, lower education level, younger age, people living with children, and people who have not been vaccinated against seasonal influenza tend to have a lower participation in follow-up. Despite the cross-country variation, the main findings are similar in the different national cohorts, and indeed the results are found to be valid also when performing a single-country analysis. Differences between countries do not seem to play a crucial role in determining the factors associated with participation in follow-up.

## Introduction

The Internet is an increasingly used tool in epidemiological data collection, especially for recruitment and follow-up of large cohorts. Successful examples of this approach include the Millennium Cohort Study [1], the Nurses and Midwives e-Cohort Study [2], the Internet-based Pregnancy Planning Study (“SnartGravid”) [3], the participatory nutritional study Nutrinet [4], and the newborn cohort NINFEA [5]. Some studies have systematically examined the advantages of the Internet-based approach [6,7], mostly in terms of the reduction of costs and time required for the data collection. Moreover, an unknown proportion of individuals with symptoms do not seek health care, and this proportion may vary with age, sex, or other social groups. Health care-seeking behavior also varies between countries and may change over the course of an epidemic. There is currently no method for systematically assessing health care usage. The use of the Internet-based cohorts can help to overcome this issue. Here we focus on Internet-based community surveillance of influenza-like-illness (ILI) as a means of gathering epidemiological data from the general population in a participatory fashion. In particular, we will study how the characteristics of individuals who register for the surveillance system are related to their involvement over the course of a surveillance season.

Influenza-like illness in Europe is monitored by means of nationwide networks of sentinel General Practitioners (GPs) detecting only medically attended ILI cases. Internet-based community surveillance of influenza can help to determine the disease burden [8], time trends and seasonality, and to characterize care-seeking and treatment behavior [9] and patterns of absenteeism [10]. Internet-based systems can thus enhance traditional GP-based surveillance and support the interpretation of recorded data, both for pandemic and seasonal influenza [8,9].

Collection of data related to influenza-like symptoms using an Internet platform is ongoing in several European countries: since 2003 in the Netherlands and Belgium [11], since 2005 in Portugal [12], 2008 in Italy, and 2009 in the United Kingdom [8,9]. Starting during the influenza season of 2011-2012, for the first time a network of Internet-based surveillance systems, called Influenzanet [13], has been active in seven European countries (the Netherlands, Belgium, Portugal, Italy, the United Kingdom, Sweden, and France), each using the same platform with the aim of gathering data across different countries in a standardized way, to monitor ILI in the community in real-time, track vaccine effectiveness [10,14], and to estimate risk factors for ILI.

In the seven countries, epidemiological data are collected with the participation of a self-selected cohort of volunteers followed over the influenza season. The success of the data collection strongly depends on the regular participation of the volunteers. Here, we investigate the determinants of participation in follow-up of volunteers enrolled in the Influenzanet platforms during the influenza season of 2011-2012.

## Methods

Any resident of a country where Influenzanet is implemented can be involved in the influenza data collection by registering through the national Web page [15-20]. In each country, identical questionnaires are implemented with questions about respiratory symptoms, access and utilization of care and self-medication, uptake of vaccines, attitudes to influenza vaccines, and absenteeism. For the sake of clarity in the following, the term “users” will indicate all the individuals who have registered with the platform and possess a username and a password, regardless of their subsequent activity during the influenza season.

Upon registration, users are invited to complete a background questionnaire containing various sociodemographic, medical, geographic, and behavioral questions. In particular, the background questionnaire covers age, gender, household size and composition, location of home and workplace, education level, occupation, vaccination status for the previous and present influenza season, the presence of a chronic disease, possible pregnancy, and other issues (see the complete list of questions in Multimedia Appendix 1). Users are reminded weekly, via an email newsletter, to report their health status through a brief symptoms questionnaire, whether or not they experienced any symptoms since the last time they visited the platform. Users who report the presence of symptoms are asked some follow-up questions about the symptoms’ onset date, about their health care-seeking behavior, medications taken for that particular episode of illness, and time off work/school. The weekly newsletter contains also a summary of the latest influenza facts and news to maintain users’ interest in the questionnaire. Users can also create accounts on behalf of other members of their family/household, thus enabling, for instance, parents to record data for their children.

To study the determinants of participation in follow-up, we analyzed the behavior of users over the course of an influenza season, focusing on their propensity to return to the platform and provide information about their health status. Information collected in the background questionnaire allows us to study how the sociodemographic characteristics of participants are related to participation in follow-up. The study sample here consisted of all the Influenzanet volunteers in the seven European countries who contributed at least one symptoms questionnaire during the 2011-2012 influenza season and who inserted their own data. Since users can also create accounts on behalf of other people of their family/household, we discarded users whose information was reported to have been provided by someone else (eg, a husband whose questionnaires are filled in by his wife). Likewise, we did not consider subjects less than 15 or more than 70 years old, on the assumption that a different participant may have managed their account. In fact, more than 400 children younger than 15 years old and more than 200 individuals older than 70 years have their own account (ie, their own username and password to access the platform). This is a legacy from previous seasons when some of the platforms had the requirement that each individual have his/her own account even if it was managed by someone else. Therefore, we did impose the additional constraint on the volunteer’s age in an attempt to ensure, as far as possible, that we were considering volunteers who manage their own accounts.

**Figure 1:**
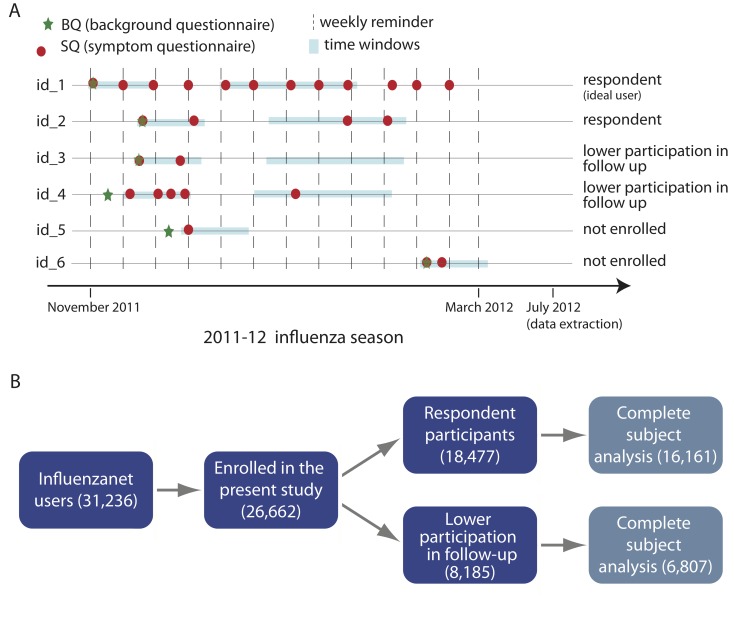
Panel A: Illustration of study definitions for enrollment and follow-up participation. Definition for enrollment: first symptoms questionnaire (SQ) at least 60 days before end of monitoring season and at least one more SQ within 15 days from first SQ; Definition for follow-up participation: at least two SQs during time window of 30 to 60 days after first SQ. Panel B: The numbers of Influenzanet users and enrolled participants, obtained applying the enrollment definition given in Panel A, are shown. The final sample on which the multilevel regression was performed, after the complete-subject exclusion of individuals with missing records, was of 22,968 enrolled individuals who provided information for all the variables. Among these, after applying the definition for follow-up participation given in Panel A, 16,161 were respondent participants and 6,807 had a lower participation in the follow-up.

From the study sample, we considered as “enrolled participants”, those participants eligible to be included in the analysis presented here, those who completed their first symptoms questionnaire at least 60 days before the end of the monitoring season, and completed at least one additional symptoms questionnaire within 15 days of the first symptoms questionnaire. From enrolled participants, we considered as “participants in follow–up” or “respondent participants” (either terms will be used in the following), those individuals who filled in at least two symptoms questionnaires during the time window of 30 to 60 days after their first symptoms questionnaire. Otherwise, the enrolled participants were considered to have a lower participation in the follow-up (see Figure 1).

In order to properly follow the unfolding of the seasonal epidemic and collect data that can be compared with the sentinel-based surveillance (which aggregates data on a weekly basis), users were asked to fill in a symptoms questionnaire each week. However, although completing the symptoms questionnaire only took a couple of minutes each week, there were numerous missing reports. Nevertheless, many individuals who missed a week did not drop out of the study; instead, they updated their health status more infrequently but they continued to provide useful information. On the other hand, some individuals registered on the website just to explore the website and its functionalities and never updated their health status; these individuals are not considered to be enrolled in the study. The definitions used for enrolled participants and participants involved in the follow-up, though by necessity somewhat arbitrary, were chosen to capture behavior that would generate a flow of useful information throughout the season.

By means of a multilevel analysis, we evaluated the association between participation in follow-up during the season and sociodemographic and behavioral characteristics collected in the background questionnaire. A multilevel analysis is generally used when the set of values of a categorical predictor variable are seen not as the complete set but rather as a random sample of all values, in order to allow inferences over a wider population than is possible with regression or other general linear model methods. In this case, we considered the follow-up participation as a dichotomous outcome, the sociodemographic and behavioral characteristics as independent variables, and the country variability as a random effect. We used as independent variables: gender, age, education level, household composition, smoking, presence of a chronic condition, and vaccination status. For each variable, we used the levels shown in Textbox 1.

**Levels used on the independent variables for the multilevel analysis.**

- gender: male, female
- age (years): 15-30, 31-40, 41-50, 51-60, 61-70
- smoking: yes (smoking occasionally or daily), no (never smoked or quit smoking)
- education level: no formal qualification (final stage of compulsory education not completed), secondary/high school education (highest level completed was the final stage of compulsory education or the high school diploma), university degree (Bachelor’s or a higher degree), and still in education (this was not a mutually exclusive category per se, as users were asked, if still in education, to select also the highest level of education achieved; we thus performed a sensitivity analysis considering this category as a missing value and obtaining very similar results)
- presence of a chronic condition/disease: yes (regularly taking medications for asthma, diabetes, lung disorder, heart disorder, kidney disorder, or an immune-compromising condition), no
- vaccination against seasonal influenza for 2011/12 season: yes, no
- household with children (≤18 years old): yes, no

In the multilevel analysis, each variable was adjusted against all the others as potential confounders for the outcome. Since some subject records for the various independent variables were missing, we used a complete-subject analysis to deal with such missing records; thus, the multilevel regression was performed only on those enrolled individuals who provided information for all the considered variables. The education level was asked only to participants above 15 years old, and therefore those with 15 years old are excluded from the multivariate analysis. The reference age group was chosen to be the one with more participants in the total population (51-60 years old) in order to reduce the estimates of confidence intervals. Given the relatively small number of clusters (ie, countries), we verified that a logistic regression where the countries are considered as dummy variables led to results comparable with those obtained with the multilevel regression in terms of strength of association (ie, odds ratios) and significance (*P* value). Data were analyzed using Stata software, version 11.0.

In the United Kingdom, the Flusurvey study was approved by the London School of Hygiene and Tropical Medicine Ethics Committee (Application number 5530). In Sweden, the Influensakoll study was approved by the Stockholm Regional Ethical Review Board (Dnr. 2011/387-31/4). In France, the Grippenet study has been approved by the Comité consultatif sur le traitement de l’information en matière de recherche (CCTIRS, Advisory committee on information processing for research, authorization 11.565), and by the Commission Nationale de l’Informatique et des Libertés (CNIL, French Data Protection Authority, authorization DR-2012-024).

In all the participating countries, the study was conducted in agreement with national regulations on privacy and data collection and treatment. Informed consent was obtained from individuals who participated in the study described in this article that the information collected through their data may be used for scientific purposes and published.

## Results

**Figure 2:**
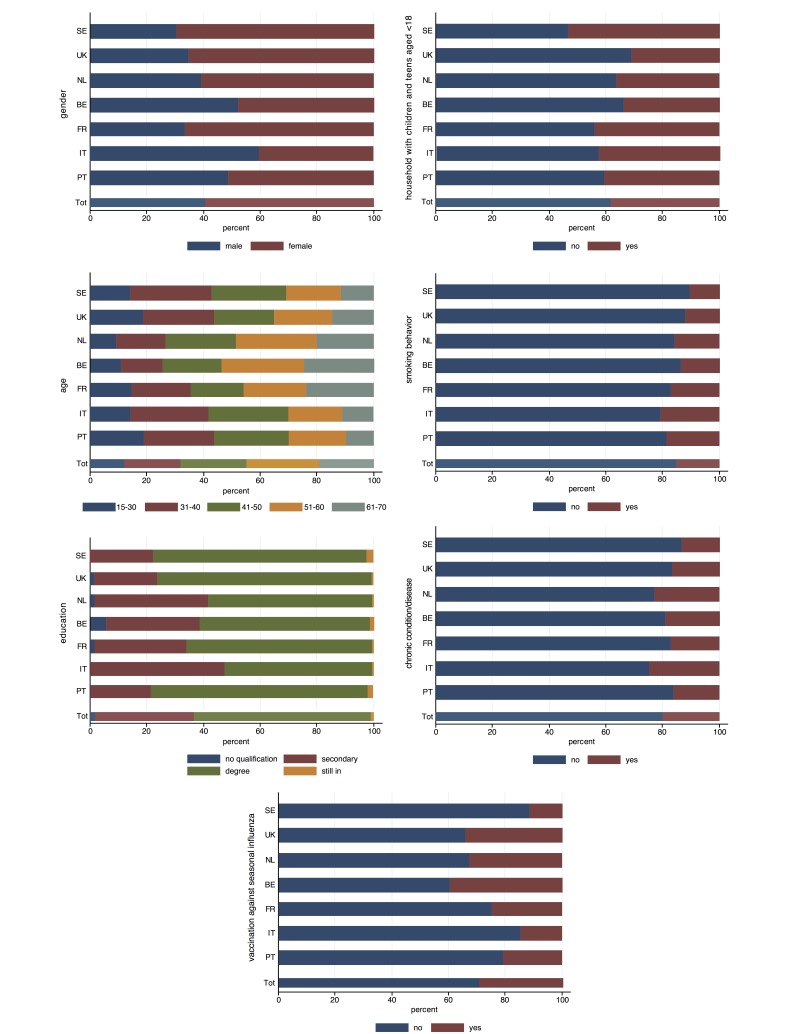
Country-specific distribution of independent variables of enrolled participants: gender, age, education level, household composition, smoking, presence of a chronic condition, and vaccination status (SE = Sweden, UK = United Kingdom, NL = the Netherlands, BE = Belgium, FR = France, IT = Italy, PT = Portugal).

Overall, 31,236 individuals registered and completed at least one symptoms questionnaire during the influenza season of 2011-2012, of whom 28,644 were aged between 15 and 70 years old, thereby comprising the sample in this study. Among these, there was a total of 26,662 enrolled participants, according to the definition above. The sociodemographic characteristics of enrolled participants are shown in Figure 2 (gender, age, education level, household composition, smoking, presence of a chronic condition, and vaccination status).

For each country, the numbers of enrolled participants and follow-up respondents are shown in Table 1. After the complete-subject exclusion of individuals with missing records, the final sample on which the multilevel regression was performed was 22,968 enrolled individuals who provided information for all the variables (86.15% of all enrolled participants, 22,968/26,662).

The proportion of respondent participants varies between 43% and 79% across the national platforms. The Dutch cohort represents almost half of the entire Influenzanet cohort (46.97%, 12,524/26,662), while the sample sizes of the others studies range between 1152 (Portugal) and 3834 (Belgium) individuals.

For comparison, we report in Table 2 the information regarding Internet access in each country (ie, the fraction of Internet users with respect to the general population).

A *P* value of <.001 associated with the likelihood-ratio test allows us to reject the null hypothesis that the between-cluster (ie, between country) variance is 0, thus confirming that a multilevel analysis is required. The results of the multilevel analysis are shown in Table 3.

The multilevel results show that men and women do not differ significantly in terms of follow- up participation. Holding a university degree, non-smoking behavior, having received an influenza vaccination, and increased age are associated with a higher participation in follow-up. Presence of a chronic health condition and living with children are associated with lower participation in follow-up.

**Figure 3:**
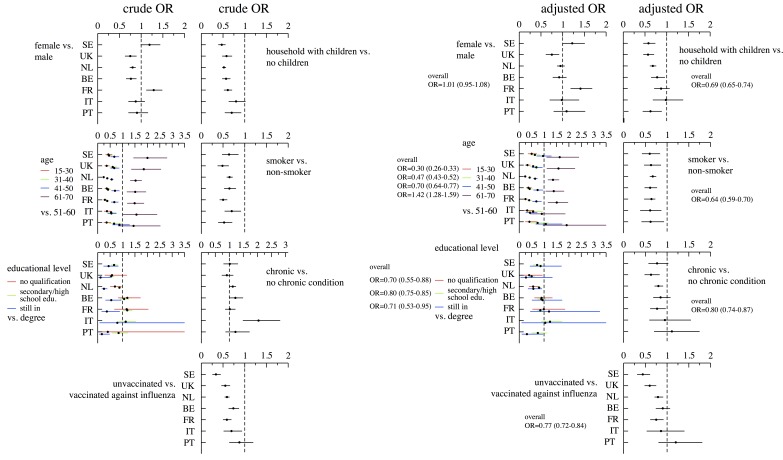
Crude (left panels) and adjusted (right panels) odds ratio by country. An odds ratio greater than 1 is associated with higher likelihood of participation in follow-up ((SE = Sweden, UK = United Kingdom, NL = the Netherlands, BE = Belgium, FR = France, IT = Italy, PT = Portugal).

We performed a separate analysis for each country by calculating both the crude and adjusted odds ratio for being a follow-up participant using the same variables that were used for the random effects multilevel analysis. Results are shown in Figure 3.

The adjusted results in Figure 3 (and Table 4 in Multimedia Appendix 2) indicate that for all the “n” countries where a significant association was obtained, participation at follow-up was positively associated with being: non-smoker (n=7 countries), older than 60 years (n=5), vaccinated against seasonal influenza (n=4), not having a chronic condition (n=3), having a university degree (vs secondary/high school, n=3; or vs having no formal education, n=2), and being female in the United Kingdom, while being male in France.

To assess the suitability of our definitions of enrollment and participation in follow-up, and to test the possible sensitivity of the results to the details of the definitions, we tested a slightly stricter definition for enrollment and follow-up participation, requiring one additional symptoms questionnaire for “enrollment”, and shifting the time window for “follow-up participation” to 45 to 75 days (instead of 30 to 60 days) after the first symptoms questionnaire. Similar conclusions were obtained for the determinants of follow-up participation when using the stricter definition for enrollment and follow-up participation (results not shown).

**Table 1:** Cohort size and participation in the seven countries considering two different enrollment definitions.

Country	Enrolled participants	Respondent participants, n (%)	Participants not involved in follow-up, n (%)
Sweden	2097	904 (43.11)	1193 (56.89)
United Kingdom	2171	1180 (54.35)	991 (45.65)
Netherlands	12,524	9679 (77.28)	2835 (22.64)
Belgium	3834	3042 (79.34)	792 (20.66)
France	3540	2227 (62.91)	1313 (37.09)
Italy	1354	657 (48.52)	697 (51.48)
Portugal	1152	788 (68.40)	364 (31.60)
Total	26,662	18,477 (69.30)	8185 (30.70)

**Table 2:** General population and fraction of Internet users for each country in the cohort.

Country	Population (2012 est.)	Internet users (30 June 2013)	Penetration (% population)
Sweden	9,103,788	8,441,718	92.73 %
United Kingdom	63,047,162	52,731,209	83.64%
Netherlands	16,730,632	15,549,787	92.94 %
Belgium	10,438,353	8,489,901	81.33 %
France	65,630,692	52,228,905	79.58 %
Italy	61,261,254	35,800,000	58.44 %
Portugal	10,781,459	5,950,449	55.19 %

**Table 3:** Random effect multilevel logistic regression performed on the set of enrolled individuals.

Variable		Reference group	Adjusted OR	95% CI	*P* value
**Gender**					
	Female	Male	1.01	0.95-1.08	.775
**Age**					
	15 - 30	51-60	0.30	0.26-0.33	<.001
	31-40	51-60	0.47	0.43-0.52	<.001
	41-50	51-60	0.70	0.64-0.77	<.001
	61 -70	51-60	1.42	1.28-1.59	<.001
**Smoking**					
	Yes	No	0.64	0.59-0.7	<.001
**Education**					
	No formal qualification	University degree	0.70	0.55-0.88	.002
	Secondary/high school education	University degree	0.80	0.75-0.85	<.001
	Still in education	University degree	0.71	0.53-0.95	.023
**Chronic condition/disease**					
	Yes	No	0.80	0.74-0.87	<.001
**Vaccination against seasonal influenza for 2011/12**					
	No	Yes	0.77	0.72-0.84	<.001
**Household with children**					
	Yes	No	0.69	0.65-0.74	<.001

## Discussion

### Principal Findings

The first year of Influenzanet data collection took place in seven European countries and saw the participation of tens of thousands of individuals among the general population. Given the opportunistic enrollment of self-selected volunteers in the Influenzanet study, it is important to investigate how sociodemographic and behavioral characteristics may be associated with follow-up participation in the Influenzanet cohort.

The study described in this paper shows that, overall, the most important determinants of participation are related to education and lifestyle: smoking (odds ratio [OR] 0.64, 95% CI 0.59-0.70), lower education level (OR ranging from 0.70 to 0.80 depending on the educational level compared with people holding a university degree), and younger age (OR ranging from 0.30 to 0.70 with an increasing trend in participation with older age) are associated with a lower rate of follow-up participation. People living with children participate in follow-up less than people living alone or in a household consisting only of adults (OR 0.66, 95% CI 0.65-0.74); people who have not been vaccinated against seasonal influenza tend to have a lower participation in follow-up with an associated (OR 0.77, 95% CI 0.72-0.84). We speculate that even though individuals living with children are at greater risk of being infected with ILI and so might be expected to be more motivated to report their health status, the demands of parental care mean that voluntary participation in follow-up could be affected by lack of time. The fact that individuals who chose to be vaccinated are more likely to be follow-up respondents could be because individuals who get the vaccine might also be individuals more concerned about their personal health and thus more likely to be involved in follow-up throughout the influenza season. Finally, males and females do not have significantly different follow-up participation behavior (OR 1.01, 95% CI 0.95-1.08).

Despite the cross-country variation, the main findings are similar in the different national cohorts, and indeed the results are found to be valid also when performing a single-country analysis. Differences between countries do not seem to play a crucial role in determining the factors associated with involvement in follow-up. The results of the multilevel analysis performed on the Influenzanet cohort as a whole indicates, as expected, that the results regarding smoking, education, and age are similar to what can be found in the literature for traditional non−Internet-based epidemiological studies [21-23] (ie, people who smoke, who are younger, and who have a lower educational level are more likely to have a lower participation in follow-up). This suggests that the self-selection of volunteers through an Internet-based system does not introduce different motivations or determinants for follow-up participation in a study with respect to traditional non−Internet-based epidemiological studies.

The overall percentage of follow-up responders for the different countries varies between 43% and 79%. The platforms running longer (the Netherlands, Belgium, and Portugal) tend to have higher proportions of follow-up responders, suggesting that the participation behavior of Influenzanet users may change over the years as they are more likely to become regular respondents the longer they are involved in the project. It is also worth mentioning that longer-standing systems may well be better able to keep people engaged, due to lessons learned about what works and what does not. Moreover, participants who continue to participate over several years are also more motivated to participate than those who drop out. In our sample, we did not distinguish between users who registered during the 2011-12 season and users from previous seasons but future work could investigate whether this explains the differences between countries. These differences could be also due to different levels of Internet penetration. The large differences in the proportion of Internet users among the general population, as shown in Table 2, can be an index of different cultural attitudes in the various countries leading to differing attitudes to participation. Further analysis might also consider the level of engagement, defining the outcome as the proportion of symptoms questionnaires completed out of the maximum possible number of questionnaires that could have been completed since registration, instead of the dichotomous definition of follow-up participation explored in the present analysis. Future work could also investigate whether the determinants associated with participation over several seasons are the same as the determinants identified in the present study.

### Conclusions

Previous studies compared the self-selected sample of Influenzanet users in a single country with the general population [8,11] and it was found that individuals recruited by the platforms are neither demographically nor geographically representative [8]. In this work, we focused on the cohort of enrolled participants from the seven countries during the 2011-12 season as a whole but we did not attempt to make comparisons with the general population. Rather, this is one of the first attempts to describe the determinants of follow-up participation in an Internet-based multi-country cohort study assessing the impact of demographic and behavioral characteristics on follow-up participation.

This study has thus provided information that will help investigators improve the planning of studies for future Internet-based surveillance and to guide enrollment and retention strategies aimed not only at enlarging the samples with motivated participants but also to enhance control over potential biases introduced by differences in follow-up behavior among participants. Our findings can additionally be used to inform the design of engagement campaigns strategically targeted at less self-motivated people to promote their participation and thereby further enhance these surveillance systems by minimizing the selection bias.

## Multimedia Appendix 1

Influenzanet questionnaires for 2011-2012.

## Multimedia Appendix 2

Factors associated with participation in the single country Influenzanet cohorts. Participation rate, crude, and adjusted odds ratios and confidence intervals are shown for different strata.

## Abbreviations

GP: General Practitioners
ILI: influenza-like-illness
OR: odds ratio
