# Supplementary material for: Determinants of Follow-Up Participation in the Internet-Based European Influenza Surveillance Platform Influenzanet
Source: J Med Internet Res. 2014 Mar 10;16(3):e78. doi: 10.2196/jmir.3010 (PMC3967126; doi:10.2196/jmir.3010)
Supplement: Supplementary file 1 [file jmir_v16i3e78_app1.PDF]

General questionnaire

Individuals who have unsubscribed from the newsletter/project or stopped reporting (no report for at least 6 weeks), should be prompted to fill in this short, separate questionnaire.

I have stopped participating because:
- *I have not had any symptoms to report*
- *I got out of the habit*
- *I do not have time*
- *The weekly questionnaire takes too long*
- *The newsletter is not interesting*
- *The website is not interesting*
- *Other reason*

Intake questionnaire

Intake Q0 (When adding another participant)
Is this person a member of your household?
- *Yes*
- *No*

Intake Q1
What is your gender?
- *Male*
- *Female*

Intake Q2
What is your date of birth?
- *Month*
- *Year*

Intake Q3
What is the first part of your home postal code?
- *Postal code*

Intake Q4
What is your main activity?
- *Paid employment, full-time*
- *Paid employment, part-time*
- *Self-employed (businessman, farmer, tradesman, etc)*
- *Attending school/college/university*
- *Home-maker (e.g. housewife)*
- *Unemployed*
- *Long-term sick-leave or parental leave*

- *Retired*
- *Other*

Intake Q4b (if Yes to Q4)
What is the first part of your school/college/workplace postal code (where you spend the majority of your working/studying time)?

- *XXXX*
- *I don't know/can't remember*
- *Not applicable (e.g. don't have a fixed workplace)*

Intake Q4c (if "Yes, Paid employment full time", or "Yes, paid employment part time" to Q4)
Which of the following descriptions most closely matches with your main occupation?

- *Professional (e.g. manager, doctor, teacher, nurse, engineer)*
- *Office work (e.g. admin, finance assistant, receptionist, etc)*
- *Retail, sales, catering and hospitality and leisure (e.g. shop assistant, waiter, bar-staff, gym instructor etc)*
- *Skilled manual worker (e.g. mechanic, electrician, technician)*
- *Other manual work (e.g. cleaning, security, driver)*
- *Other (Free text field if "other" checked, allows the user to write in their occupation)*

Intake Q4d (to be asked for those aged 16 and over)
What is the highest level of formal education qualification that you have? If you are still in education, then please tick this box with the appropriate highest level that you have already achieved.

- *I have no formal qualifications*
- *GCSE's, O'levels, CSEs or equivalent*
- *A-Levels or equivalent (e.g. Highers, NVQ Level3, BTEC)*
- *Batchelors Degree (BA, BSc) or equivalent (e.g. HND, NVQ Level 4)*
- *Higher Degree or equivalent (e.g. Masters Degree, PGCE, PhD, Medical Doctorate, Advanced Professional Awards)*
- *I am still in education*

Intake Q5
Do you have contact with any of the following during the course of a typical day? (Select all options that apply, if any)

- *More than 10 children or teenagers over the course of the day*
- *More than 10 people aged over 65 over the course of day*
- *Patients*
- *Groups of people (more than 10 individuals at any one time)*
- *None of the above*

Intake Q6
Including you, how many people in each of the following age groups live in your house-hold?

- *0-4 years*

- *5-18 years*
- *19-44 years*
- *45-64 years*
- *65+ years*

Intake Q6b (If any in household are aged 0-4, including participant)
How many of the children aged 0-4 in your household go to school or day-care?

- *Drop-down menu*

Intake Q7
What is your main means of transportation?

- *Walking*
- *Bike*
- *Motorbike/scooter*
- *Car*
- *Public transportation (bus, train, tube, etc)*
- *Other*

Intake Q7b
On a normal day, how much time do you spend on public transport (bus, train, tube, etc)?

- *No time at all*
- *0 - 30 minutes*
- *30 minutes - 1.5 hours*
- *1.5 hours - 4 hours*
- *Over 4 hours*

Intake Q8
How often do you have common colds or flu-like diseases?

- *Never*
- *Once or twice a year*
- *Between 3 and 5 times a year*
- *Between 6 and 10 times a year*
- *More than 10 times a year*
- *I don't know*

Intake Q9
Did you receive a flu vaccine during the last autumn/winter season (i.e. 2010-2011)?

- *Yes*
- *No*
- *I don't know/can't remember*

Intake Q10
Have you received a flu vaccine this autumn/winter season (2011-2012)?

- *Yes [go to questions Q10b and Q10c]*
- *No [go to question Q10d]*
- *I don't know/can't remember*

© Influenzanet - Intake and Weekly symptoms' questionnaire, UK version 110901.

Intake Q10b: Follow-up question
When were you vaccinated against flu this season (2011-2012)?
- *Choose date: X/XX/XXXX*
- *I don't know/can't remember*

Intake Q10c: Follow-up question
What were your reasons for getting a seasonal influenza vaccination this year? (Select all options that apply)
- *I belong to a risk group (e.g. pregnant, over 65, underlying health condition, etc)*
- *Vaccination decreases my risk of getting influenza*
- *Vaccination decreases the risk of spreading influenza to others*
- *My doctor recommended it*
- *It was recommended in my workplace/school*
- *The vaccine was readily available and vaccine administration was convenient*
- *The vaccine was free (no cost)*
- *I don't want to miss work/school*
- *I always get the vaccine*
- *Other reason(s)*

Intake Q10d: Follow-up question
What were your reasons for NOT getting a seasonal influenza vaccination this year? (Select all options that apply)
- *I am planning to be vaccinated, but haven't been yet*
- *I haven't been offered the vaccine*
- *I don't belong to a risk group*
- *It is better to build your own natural immunity against influenza*
- *I doubt that the influenza vaccine is effective*
- *Influenza is a minor illness*
- *I don't think that I am likely to get influenza*
- *I believe that influenza vaccine can cause influenza*
- *I am worried that the vaccine is not safe or will cause illness or other adverse events*
- *I don't like having vaccinations*
- *The vaccine is not readily available to me*
- *The vaccine is not free of charge*
- *No particular reason*
- *Although my doctor recommended a vaccine, I did not get one*
- *Other reason(s)*

Intake Q11
Do you take regular medication for any of the following medical conditions? (Select all options that apply)
- *No*
- *Asthma*
- *Diabetes*
- *Lung disorder (COPD, emphysema, …)*
- *Heart disorder*

- *Kidney disorder*
- *An immunocompromising condition (e.g. splenectomy, organ transplant, acquired immune deficiency, cancer treatment)*

Intake Q12 (only asked of women between ages 15 and 50)
Are you currently pregnant?
- *Yes [go to question Q12b]*
- *No*
- *Don't know/would rather not answer*

Intake Q12b: follow-up question
Which trimester of the pregnancy are you in?
- *First trimester (week 1-12)*
- *Second trimester (week 13-28)*
- *Third trimester (week 29-delivery)*
- *Don't know/would rather not answer*

Intake Q13
Do you smoke tobacco?
- *No*
- *Yes, occasionally*
- *Yes, daily, fewer than 10 times a day*
- *Yes, daily, 10 or more times a day*
- *Don't know/would rather not answer*

Intake Q14
Do you have one of the following allergies that can cause respiratory symptoms? (Select all options that apply)
- *Hay fever*
- *Allergy against house dust mite*
- *Allergy against domestic animals or pets*
- *Other allergies that cause respiratory symptoms (e.g. sneezing, runny eyes)*
- *I do not have an allergy that causes respiratory symptoms*

Intake Q15 (Not a core question)
Do you follow a special diet? (Select all options that apply)
- *No special diet*
- *Vegetarian*
- *Veganism*
- *Low-calorie*
- *Other*

Intake Q16 (Not a core question)
Do you have pets at home? (Select all options that apply)
- *No*
- *Yes, one or more dogs*

- *Yes, one or more cats*
- *Yes, one or more birds*
- *Yes, one ore more other animals*

Symptoms questionnaire

Weekly Q1
Have you had any of the following symptoms since your last visit (or in the past week, if this is your first visit)? (Select all options that apply)
- *No symptoms*
- *Fever*
- *Chills*
- *Runny or blocked nose*
- *Sneezing*
- *Sore throat*
- *Cough*
- *Shortness of breath*
- *Headache*
- *Muscle/joint pain*
- *Chest pain*
- *Feeling tired or exhausted*
- *Loss of appetite*
- *Coloured sputum/phlegm*
- *Watery, bloodshot eyes*
- *Nausea*
- *Vomiting*
- *Diarrhoea*
- *Stomach ache*
- *Other*

Weekly Q2
(If the participant was STILL ILL on their last visit and has reported symptoms this time):
"On DATE OF LAST VISIT you reported that you were still ill with symptoms that began on DATE OF FIRST SYMPTOMS REPORTED PREVIOUSLY. Are the symptoms you reported today part of the same bout of illness?
- *Yes*
- *No*
- *I don't know/can't remember*

If NO or DON'T KNOW: [This means that the current symptoms are the start of a "new" illness: continue with Weekly Q3]

if YES: [This means that the current symptoms are the continuation of the same bout of illness as the previous visit. The remainder of the symptoms questionnaire (onset date, further details about symptoms, details about seeking medical attention, treatment, time off

work/school) can be pre-filled with their previous answers:]

To save you time, we have filled in the information you gave us previously about your illness. Please check that it is still correct, and make any changes – for instance, if you have visited a doctor or taken additional time off work since you last completed the survey.

Weekly Q3 (if symptoms)
When did the first symptoms appear?
- *Choose date XX/XX/XXXX*
- *I don't know/can't remember*

Weekly Q4 (if symptoms)
When did your symptoms end?
- *Choose date XX/XX/XXXX*
- *I don't know/can't remember*
- *I am still ill*

Weekly Q5 (if symptoms)
Did your symptoms develop suddenly over a few hours?
- *Yes*
- *No*
- *I don't know*

Weekly Q6 (if fever)
When did your fever begin?
- *Choose date XX/XX/XXXX*
- *I don't know/can't remember*

Weekly Q6a (if fever, not a core question)
Did you take your temperature?
- *Yes*
- *No*
- *I don't know*

Weekly Q6b (if symptoms)
Did you take your temperature?
- *Yes [go to Weekly Q6c]*
- *No*
- *I don't know*

Weekly Q6c (if symptoms) and (if took temperature): follow-up question
What was the highest temperature measured?
- *Below 37°*
- *37° - 37.4°C*
- *37.5° - 37.9°C*
- *38° - 38.9°C*

- *39° - 39.9°C*
- *40°C or more*
- *I don’t know/can’t remember*

Weekly Q7 (if symptoms)
Because of your symptoms, did you VISIT (see face to face) any of the following? (Select all options that apply)
- *No*
- *GP or GP’s practice nurse*
- *Hospital admission*
- *Hospital accident & emergency department/out of hours service*
- *Other medical services*
- *No, but I have an appointment scheduled*

Weekly Q7b (if symptoms)
How soon after your symptoms appeared did you visit this medical service?
- *Same day*
- *1 day*
- *2 days*
- *3 days*
- *4 days*
- *5-7 days*
- *More than 7 days*
- *I don’t know/can’t remember*

Weekly Q8 (if symptoms)
Because of your symptoms, did you contact via TELEPHONE or INTERNET any of the following? (Select all options that apply)
- *No*
- *GP - spoke to receptionist only*
- *GP - spoke to doctor or nurse*
- *NHS Direct / NHS 24 / NHS Choices*
- *NPFS*
- *Other*

Weekly Q8b (if symptoms)
How soon after your symptoms appeared did you contact via telephone or internet any of the following?
- *Same day*
- *1 day*
- *2 days*
- *3 days*
- *4 days*
- *5-7 days*
- *More than 7 days*
- *I don’t know/can’t remember*

Weekly Q9 (if symptoms)
Did you take medication for these symptoms? (Select all options that apply)
- *No medication*
- *Pain killers or antipyretics (e.g. paracetamol, lemsip, ibuprofen, aspirin, calpol, etc)*
- *Cough medication (e.g. expectorants)*
- *Antivirals (Tamiflu, Relenza)*
- *Antibiotics*
- *Other*
- *I don’t know/can’t remember*

Weekly Q9b (if antivirals were taken): follow-up question
How long after the beginning of your symptoms did you start taking antiviral medication?
- *Same day (within 24 hours)*
- *1 day later*
- *2 days later*
- *3 days later*
- *4 days later*
- *5-7 days later*
- *More than 7 days later*
- *I don’t know/can’t remember*

Weekly Q10 (if symptoms)
Did you change your daily routine because of your illness?
- *No*
- *Yes, but I did not take time off work/school*
- *Yes, I took time off work/school*

Weekly Q10b (if symptoms) & (if taken time off work/school): follow-up question
Are you still off work/school?
- *Yes*
- *No*
- *Other (e.g. I wouldn’t usually be at work/school today anyway)*

Weekly Q10c (if symptoms) & (if taken time off work/school): follow-up question
How long have you been off work/school for?
- *1 day*
- *2 days*
- *3 days*
- *4 days*
- *5 days*
- *6 to 10 days*
- *11 to 15 days*
- *More than 15 days*

Weekly Q11 (if symptoms, not a core question)
What do you think is causing your symptoms?

- *Flu or flu-like illness*
- *Common cold*
- *Allergy/hay fever*
- *Gastroenteritis/gastric flu*
- *Other*
- *I don't know*

Weekly Q12
How many members of your household have had flu-like symptoms in the past week?
- *0*
- *1*
- *2*
- *3*
- *4*
- *5*
- *6 or more*
- *I don't know*

Weekly Q13
Excluding household members, how many people did you meet with flu-like symptoms in the past week?
- *0*
- *1*
- *2*
- *3*
- *4*
- *5*
- *6 or more*
- *I don't know*
