# Supplementary material for: Determinants of Follow-Up Participation in the Internet-Based European Influenza Surveillance Platform Influenzanet
Source: J Med Internet Res. 2014 Mar 10;16(3):e78. doi: 10.2196/jmir.3010 (PMC3967126; doi:10.2196/jmir.3010)
Supplement: Supplementary file 2 [file jmir_v16i3e78_app2.pdf]

Table 4: Factors associated with the participation in the single country Influenzanet cohorts. Participation rate, crude and adjusted odds ratios and confidence intervals are shown for different strata.

**Sweden** (www.influensakoll.se)						
**Variable (target population)**	**Respondent participants (%)**	**Participants not involved in follow-up (%)**	**OR (crude)**	**95% C.I.**	**OR (adjusted)**	**95% C.I.**
Gender (2097)						
Male (30%)	256 (40)	382 (60)	1.00		1.00	
Female (70%)	648 (44)	811 (56)	1.19	0.99-1.44	1.22	0.99-1.51
Age (2097)						
15 - 30 (14%)	95 (32)	203 (68)	0.43	0.31-0.59	0.50	0.35-0.71
31 - 40 (29%)	199 (33)	400 (67)	0.46	0.35-0.59	0.63	0.46-0.85
41 - 50 (26%)	234 (42)	322 (58)	0.67	0.51-0.86	0.96	0.70-1.30
51 - 60 (19%)	208 (52)	191 (48)	1.00		1.00	
61 - 70 (12%)	168 (69)	77 (31)	2.0	1.43-2.8	1.63	1.10-2.41
Smoking (2096)						
No (89%)	828 (44)	1045 (56)	1.00		1.00	
Yes (11%)	75 (34)	148 (66)	0.64	0.48-0.86	0.60	0.42-0.84
Education (1903)						
No formal qualification (<1%)	1 (100)	0 (0)	-	-	-	-
Secondary/high school education (22%)	151 (36)	271 (64)	0.66	0.53-0.82	0.72	0.56-0.92
Still in education (3%)	13 (27)	35 (73)	0.44	0.23-0.84	0.86	0.43-1.71
University Degree (75%)	656 (46)	776 (54)	1.00		1.00	
Chronic condition/disease (2097)						
No (86%)	781 (43)	1033 (57)	1.00		1.00	
Yes (12%)	123 (43)	160 (57)	1.02	0.79-1.31	0.77	0.57-1.03
Vaccination against seasonal influenza for 2011/12 (2094)						
Yes (12%)	161 (67)	81 (33)	1.00		1.00	
No (88%)	743 (40)	1109 (60)	0.34	0.25-0.45	0.44	0.31-0.61
Household with children (2009)						

No (47%)	495 (53)	442 (47)	1.00		1.00	
Yes (53%)	372 (35)	700 (65)	0.47	0.39-0.56	0.58	0.46-0.74
**United Kingdom** (www.flusurvey.org.uk)						
**Variable (target population)**	**Respondent participants (%)**	**Participants not involved in follow-up (%)**	**OR (crude)**	**95% C.I.**	**OR (adjusted)**	**95% C.I.**
Gender (2171)						
Male (35%)	442 (59)	307 (41)	1.00		1.00	
Female (65%)	738 (52)	684 (48)	0.87	0.7-1.09	0.75	0.61-0.92
Age (2171)						
15 - 30 (19%)	152 (37)	256 (63)	0.36	0.27-0.47	0.33	0.23-0.45
31 - 40 (25%)	271 (50)	271 (50)	0.60	0.47-0.78	0.56	0.42-0.74
41 - 50 (21%)	239 (52)	217 (48)	0.67	0.51-0.87	0.69	0.51-0.94
51 - 60 (20%)	279 (62)	169 (38)	1.00		1.00	
61 - 70 (15%)	239 (75)	78 (25)	1.86	1.35-2.55	1.58	1.11-2.25
Smoking (2164)						
No (88%)	1078 (57)	824 (43)	1.00		1.00	
Yes (12%)	102 (39)	160 (61)	0.49	0.37-0.63	0.63	0.47-0.86
Education (2157)						
No formal qualification (1%)	14 (45)	17 (55)	0.58	0.28-1.18	0.40	0.17-0.94
Secondary/High school education (22%)	209 (43)	275 (57)	0.55	0.44-0.67	0.50	0.39-0.64
Still in education (1%)	2 (14)	12 (86)	0.12	0.03-0.52	0.27	0.05-1.34
University Degree (75%)	950 (58)	678 (42)	1.00		1.00	
Chronic condition/disease (2171)						
No (83%)	988 (55)	817 (45)	1.00		1.00	
Yes (17%)	192 (52)	174 (48)	0.91	0.73-1.14	0.63	0.48-0.83
Vaccination against seasonal influenza for 2011/12 (2153)						
Yes (34%)	475 (64)	264 (36)	1.00		1.00	
No (66%)	705 (50)	709 (50)	0.55	0.46-0.66	0.60	0.48-0.75
Household with children (2069)						
No (69%)	828 (58)	599 (42)	1.00		1.00	
Yes (31%)	288 (45)	354 (55)	0.58	0.48-0.71	0.57	0.46-0.71

**The Netherlands** (www.degrotegriepmeting.nl)						
**Variable (target population)**	**Respondent participants (%)**	**Participants not involved in follow-up (%)**	**OR (crude)**	**95% C.I.**	**OR (adjusted)**	**95% C.I.**
Gender (12514)						
Male (39%)	3891 (80)	994 (20)	1.00		1.00	
Female (61%)	5788 (76)	1841 (24)	0.8	0.74-0.88	0.95	0.87-1.05
Age (12514)						
15 - 30 (9%)	651 (56)	509 (44)	0.24	0.21-0.28	0.24	0.20-0.29
31 - 40 (17%)	1448 (67)	707 (33)	0.38	0.34-0.44	0.42	0.36-0.49
41 - 50 (25%)	2325 (75)	777 (25)	0.56	0.5-0.63	0.64	0.56-0.74
51 - 60 (28%)	3000 (84)	564 (16)	1.00		1.00	
61 - 70 (20%)	2255 (89)	278 (11)	1.52	1.31-1.78	1.36	1.15-1.61
Smoking (12493)						
No (84%)	8257 (79)	2239 (21)	1.00		1.00	
Yes (16%)	1409 (71)	588 (29)	0.65	0.58-0.72	0.67	0.60-0.75
Education (12277)						
No formal qualification (1%)	133 (72)	51 (28)	0.69	0.5-0.96	0.56	0.39-0.81
Secondary/high school education (40%)	3758 (76)	1164 (24)	0.87	0.8-0.96	0.80	0.73-0.89
Still in education (1%)	38 (49)	40 (51)	0.25	0.16-0.39	0.57	0.35-0.92
University Degree (58%)	5579 (79)	1514 (21)	1.00		1.00	
Chronic condition/disease (12514)						
No (77%)	7407 (77)	2226 (23)	1.00		1.00	
Yes (23%)	2272 (79)	609 (21)	1.12	1.01-1.24	0.80	0.71-0.91
Vaccination against seasonal influenza for 2011/12 (12506)						
Yes (33%)	3402 (83)	687 (17)	1.00		1.00	
No (67%)	6273 (64)	2144 (25)	0.59	0.54-0.65	0.81	0.71-0.91
Household with children (12146)						
No (64%)	6296 (82)	1420 (18)	1.00		1.00	
Yes (36%)	3101 (70)	1329 (30)	0.52	0.48-0.57	0.68	0.61-0.76
**Belgium**						

(www.degrotegriepmeting.be)						
**Variable (target population)**	**Respondent participants (%)**	**Participants not involved in follow-up (%)**	**OR (crude)**	**95% C.I.**	**OR (adjusted)**	**95% C.I.**
Gender (3834)						
Male (52%)	1626 (81)	370 (19)	1.00		1.00	
Female (48%)	1416 (77)	422 (23)	0.76	0.65-0.89	0.92	0.77-1.09
Age (3834)						
15 - 30 (11%)	271 (65)	146 (35)	0.36	0.28-0.47	0.37	0.27-0.50
31 - 40 (14%)	382 (68)	180 (32)	0.42	0.33-0.53	0.42	0.32-0.56
41 - 50 (21%)	621 (78)	175 (22)	0.7	0.55-0.88	0.74	0.57-0.96
51 - 60 (29%)	928 (83)	182 (16)	1.00		1.00	
61 - 70 (25%)	840 (89)	109 (11)	1.51	1.17-1.95	1.39	1.06-1.81
Smoking (3831)						
No (86%)	2657 (80)	648 (20)	1.00		1.00	
Yes (14%)	382 (73)	144 (27)	0.65	0.52-0.8	0.61	0.48-0.77
Education (3688)						
No formal qualification (6%)	166 (82)	37 (18)	1.19	0.82-1.73	0.90	0.61-1.34
Secondary/high school education (33%)	970 (80)	244 (20)	1.06	0.89-1.27	0.89	0.74-1.08
Still in education (1%)	39 (67)	19 (33)	0.54	0.31-0.95	0.94	0.51-1.73
University Degree (60%)	1747 (79)	466 (21)	1.00		1.00	
Chronic condition/disease (3834)						
No (81%)	2434 (79)	657 (21)	1.00		1.00	
Yes (19%)	608 (82)	135 (18)	1.22	0.99-1.49	0.85	0.68-1.08
Vaccination against seasonal influenza for 2011/12 (3829)						
Yes (40%)	1249 (82)	269 (18)	1.00		1.00	
No (60%)	1789 (77)	522 (23)	0.74	0.63-0.87	0.90	0.74-1.07
Household with children (3715)						
No (66%)	2031 (83)	428 (17)	1.00		1.00	
Yes (34%)	918 (73)	338 (27)	0.57	0.48-0.67	0.78	0.64-0.96
**France** (www.grippenet.fr)						
**Variable (target**	**Respondent**	**Participants**	**OR**	**95% C.I.**	**OR**	**95% C.I.**

population)	participants (%)	not involved in follow-up (%)	(crude)		(adjusted)	
Gender (3540)						
Male (33.5%)	699 (59)	487 (41)	1.00		1.00	
Female (66.5%)	1528 (65)	826 (35)	1.29	1.12-1.49	1.41	1.18-1.68
Age (3540)						
15 - 30 (15%)	221 (42)	301 (58)	0.29	0.23-0.36	0.25	0.18-0.34
31 - 40 (20%)	361 (49)	369 (51)	0.38	0.31-0.48	0.40	0.31-0.53
41 - 50 (19%)	422 (63)	248 (37)	0.67	0.54-0.83	0.69	0.53-0.90
51 - 60 (22%)	557 (72)	219 (28)	1.00		1.00	
61 - 70 (24%)	666 (79)	176 (21)	1.49	1.18-1.87	1.51	1.15-1.97
Smoking (3533)						
No (83%)	1925 (66)	1003 (34)	1.00		1.00	
Yes (17%)	297 (49)	308 (51)	0.5	0.42-0.6	0.59	0.47-0.73
Education (3453)						
No formal qualification (2%)	37 (66)	19 (34)	1.17	0.67-2.04	0.99	0.53-1.85
Secondary/high school education (32%)	721 (65)	392 (35)	1.19	1.02-1.39	0.85	0.71-1.03
Still in education (1%)	8 (38)	13 (62)	0.36	0.15-0.89	1.2	0.44-3.25
University Degree (66%)	1412 (62)	851 (38)	1.00		1.00	
Chronic condition/disease (3540)						
No (83%)	1845 (63)	1091 (37)	1.00		1.00	
Yes (17%)	382 (63)	222 (37)	1.02	0.85-1.22	0.77	0.61-0.97
Vaccination against seasonal influenza for 2011/12 (3535)						
Yes (25%)	633 (72)	248 (28)	1.00		1.00	
No (75%)	1593 (60)	1061 (40)	0.59	0.5-0.69	0.75	0.61-0.92
Household with children (2945)						
No (56%)	1124 (68)	528 (32)	1.00		1.00	
Yes (44%)	733 (57)	560 (43)	0.61	0.52-0.71	0.87	0.71-1.07
**Italy** (www.influweb.it)						
**Variable (target population)**	**Respondent Participants**	**Participants not involved**	**OR (crude)**	**95% C.I.**	**OR (adjuste**	**95% C.I.**

	(%)	in follow-up (%)			d)	
Gender (1354)						
Male (60%)	402 (50)	404 (50)	1.00		1.00	
Female (40%)	255 (47)	293 (53)	0.87	0.7-1.09	0.98	0.70-1.38
Age (1354)						
15 - 30 (14%)	68 (35)	126 (65)	0.37	0.25-0.55	0.32	0.17-0.60
31 - 40 (28%)	164 (44)	209 (56)	0.54	0.39-0.74	0.56	0.33-0.95
41 - 50 (28%)	169 (44)	211 (56)	0.55	0.4-0.76	0.43	0.26-0.73
51 - 60 (19%)	154 (59)	106 (41)	1.00		1.00	
61 - 70 (11%)	102 (69)	45 (31)	1.56	1.02-2.39	0.91	0.44-1.86
Smoking (1344)						
No (79%)	537 (50)	530 (50)	1.00		1.00	
Yes (21%)	115 (42)	162 (58)	0.70	0.54-0.91	0.57	0.38-0.87
Education (693)						
No formal qualification (0%)	0	0	-	-	-	-
Secondary/high school education (47%)	137 (42)	192 (58)	1.13	0.83-1.54	1.24	0.89-1.73
Still in education (1%)	1 (33)	2 (66)	0.79	0.07-8.8	1.05	0.09-12.13
University Degree (52%)	136 (38)	225 (62)	1.00		1.00	
Chronic condition/disease (823)						
No (75%)	239 (39)	381 (62)	1.00		1.00	
Yes (25%)	114 (56)	89 (44)	2.04	1.48-2.81	0.95	0.59-1.55
Vaccination against seasonal influenza for 2011/12 (1354)						
Yes (15%)	112 (56)	87 (44)	1.00		1.00	
No (85%)	545 (47)	610 (53)	0.69	0.51-0.94	0.86	0.53-1.4
Household with children (1069)						
No (57%)	282 (46)	329 (54)	1.00		1.00	
Yes (43%)	187 (41)	271 (59)	0.80	0.63-1.02	0.98	0.68-1.38
**Portugal** (www.gripenet.pt)						
**Variable (target population)**	**Respondent Participants (%)**	**Participants not involved in follow-up (%)**	**OR (crude)**	**95% C.I.**	**OR (adjusted)**	**95% C.I.**
Gender (1152)						

Male (49%)	390 (70)	171 (30)	1.00		1.00	
Female (51%)	398 (67)	193 (33)	0.90	0.7-1.16	1.09	0.79-1.52
Age (1152)						
15 - 30 (19%)	113 (52)	105 (48)	0.36	0.24-0.53	0.41	0.24-0.71
31 - 40 (25%)	190 (66)	96 (34)	0.66	0.45-0.97	0.74	0.46-1.18
41 - 50 (26%)	220 (72)	84 (28)	0.87	0.59-1.29	1.07	0.66-1.73
51 - 60 (20%)	174 (75)	58 (25)	1.00		1.00	
61 - 70 (10%)	91 (81)	21 (19)	1.44	0.82-2.53	1.91	0.95-3.83
Smoking (1149)						
No (81%)	666 (71)	269 (29)	1.00		1.00	
Yes (19%)	121 (57)	93 (43)	0.52	0.39-0.71	0.62	0.41-0.93
Education (931)						
No formal qualification (1%)	1 (50)	1 (50)	0.40	0.02-6.5	-	-
Secondary/high school education (21%)	124 (62)	75 (38)	0.84	0.58-1.22	0.75	0.50-1.13
Still in education (2%)	5 (29)	12 (71)	0.17	0.06-0.48	0.31	0.10-0.96
University Degree (77%)	502 (70)	211 (30)	1.00		1.00	
Chronic condition/disease (1152)						
No (84%)	655 (68)	312 (32)	1.00		1.00	
Yes (16%)	133 (72)	52 (28)	1.22	0.86-1.72	1.11	0.70-1.75
Vaccination against seasonal influenza for 2011/12 (1149)						
Yes (21%)	169 (71)	70 (29)	1.00		1.00	
No (79%)	618 (68)	292 (32)	0.88	0.64-1.2	1.20	0.80-1.81
Household with children (1067)						
No (59%)	453 (71)	181 (29)	1.00		1.00	
Yes (41%)	277 (64)	156 (36)	0.70	0.54-0.92	0.61	0.43-0.87
